# Population structure and genetic variation of cucumber mosaic virus isolates in Serbia: evidence for high diversity and the occurrence of natural recombinant and reassortant isolates

**DOI:** 10.3389/fpls.2026.1741618

**Published:** 2026-02-19

**Authors:** Katarina Zečević, Smilja Teodorović, Ana Vučurović, Branka Krstić, Dušica Kovačević, Ivana Stanković

**Affiliations:** 1 Institute of Phytomedicine, Department of Phytopathology, University of Belgrade-Faculty of Agriculture, Belgrade, Serbia; 2 Department of Forensic Sciences, University of Criminal Investigation and Police Studies, Belgrade, Serbia; 3 Department of Biotechnology and Systems Biology, National Institute of Biology, Ljubljana, Slovenia

**Keywords:** CMV, genetic diversity, molecular characterization, population genetic parameters, recombination and reassortment

## Abstract

Cucumber mosaic virus (CMV) causes significant economic losses and threatens the sustainable production of many important crops. This study represents the first analysis of CMV genetic diversity and population structure in Serbia, based on all five ORFs from nineteen isolates collected across various hosts and regions. Results of molecular and population genetic investigations revealed high genetic diversity and the presence of numerous genetic variants. Phylogenetic analyses showed that isolates from all three subgroups exist in Serbia, with IA being the most prevalent and IB occurring at the 1a gene only. Although findings indicate that majority of the observed variation arises from differences between subgroups, intra-subgroup fixed polymorphisms were also identified. Notably, both recombinants and reassortants were detected, indicating dynamic genetic exchange within the population. Recombination between subgroups IA and II was confirmed in both RNA 2 (II-IA,II-II,II and IB-IA,II-IA,IA) and RNA 3 segments (IA-IA,IA-IA,II), with recombination in the RNA 2 segment being predominant. Additionally, both types of reassortment, including IA/IB (IB-IA,IA-IA,IA) and IA/II (II-IA,IA-II,II) reassortants, were recorded. Moreover, we uncover a novel reassortant/recombinant variant (IB-IA,II-IA,IA) in the natural CMV population. Finally, results of a neutrality test across all five loci suggest demographic effects consistent with population size expansion following bottleneck events during host plant infection. This study provides the first molecular insight into CMV population structure in Serbia, offering new insights into the regional diversity and evolution of the virus. By identifying recombinant and reassortant CMV isolates circulating in Serbia, this work reveals previously unrecognized evolutionary processes shaping CMV populations in the region. These findings fill a significant geographic gap in CMV research in Western Balkans and contribute to a broader understanding of CMV evolution, diversification, and population dynamics globally.

## 1 Introduction

Cucumber mosaic virus (CMV; species Cucumovirus CMV, genus Cucumovirus, family Bromoviridae) is one of the most economically important plant viruses causing major losses in many agricultural crops worldwide. It probably has one of the broadest host ranges among plant viruses, covering 1,300 different plant species in 500 genera from more than 100 families (García-Arenal and Palukaitis, 2008; Ohshima et al., 2016; Hirsch and Moury, 2021). CMV is primarily transmitted by more than 80 species of aphids in a non-persistent manner, but it is also mechanically transmissible by plant sap, through seeds of some plant species and parasitic plants (García-Arenal and Palukaitis, 2008). In addition, its spread in nature is facilitated by the infection of various wild and weed species that serve as reservoirs (Palukaitis et al., 1992; Gallitelli, 2000; Gildow et al., 2008; Jacquemond, 2012).

CMV genome consists of three ss (+) RNAs, designated RNA 1, RNA 2 and RNA 3, which encode five proteins. Proteins 1a and 2a are encoded by RNA 1 and RNA 2, respectively, and form the RNA-dependent RNA polymerase viral component of the replicase complex (Roossinck, 2002). Protein 2b, also encoded by RNA 2, is involved in host-specific long-distance movement, symptom severity, and suppression of RNA silencing. Proteins 3a (movement protein, MP) and 3b (capsid protein, CP) are encoded by the bicistronic RNA 3. MP is involved in cell-to-cell and vascular movement of the virus, as well as aphid-mediated transmission, while CP participates in several processes, including virion assembly, host range, aphid transmission, and both cell-to-cell and systemic movement (Palukaitis et al., 1992; Roossinck, 2002; Jacquemond, 2012). In addition, some CMV isolates are associated with satellite RNAs (satRNAs), non-coding transcripts that can alter the symptoms of CMV infections (Palukaitis et al., 1992).

Phylogenetically, numerous CMV isolates are classified into three groups named subgroup IA, IB and II (Palukaitis et al., 1992; Rizos et al., 1992; Wahyuni et al., 1992; Roossinck et al., 1999). Subgroups IA and II are distributed worldwide, whereas subgroup IB is believed to have originated in Asia, although it has also been found in other regions. In Europe, subgroup IB has so far been reported only in Spain, Italy, Greece, and France (Lin et al., 2003; Palukaitis and García-Arenal, 2003; Eiras et al., 2004; Bonnet et al., 2005; Sclavounos et al., 2006; García-Arenal and Palukaitis, 2008; Jacquemond, 2012; Kayode et al., 2014; Giakountis et al., 2018; Gaudin et al., 2025). However, frequent genetic exchange through recombination and reassortment may lead to discrepancies in this classification by creating novel genetic combinations and hybrid genomes that may not fit into established subgroups (Ohshima et al., 2016). Numerous studies investigating the genetic structure of natural CMV populations, focusing either on specific open reading frames (ORFs) or, more recently, on complete genomes, have pointed out the presence of recombinant and reassortant isolates across the world (Chen et al., 2007; Liu et al., 2009; Maoka et al., 2010; Jacquemond, 2012; Ben Tamarzizt et al., 2013; Kim et al., 2014; Nouri et al., 2014; Hasiów-Jaroszewska et al., 2017, 2018). Moreover, in some countries (e.g., Tunisia), such isolates have become well established and predominant within local population (Ben Tamarzizt et al., 2013).

In Serbia, CMV has been identified as one of the most economically important plant viruses due to its wide host range, efficient aphid-mediated transmission, and capacity to cause substantial yield and quality losses. The virus poses major threats to the production of various vegetable, field, and ornamental crops, including tomato (Nikolić et al., 2018; Stanković et al., 2021), pepper (Milošević et al., 2017), tobacco (Stanković et al., 2011), and cucurbits (Vučurović et al., 2011; Milojević et al., 2013). Given Serbia’s central geographic location and its role as an important agricultural hub within the Western Balkans, CMV populations circulating in the country may serve as a key source of virus dissemination throughout the region. The high genetic variability of CMV, driven by mutation, reassortment, and recombination, facilitates rapid adaptation to diverse hosts and environments, posing significant challenges to disease management not only in Serbia but across the Western Balkans. Consequently, improved knowledge of CMV diversity and population structure in Serbia is essential for understanding its epidemiology and evolution at the regional level.

Although genetic variability and epidemiology of CMV have been studied in several parts of the world, including the USA (Lin et al., 2004; Nouri et al., 2014) and Spain (Fraile et al., 1997; Bonnet et al., 2005), detailed molecular data on Serbian isolates remain limited. Previous studies have focused on its occurrence and associated damage, while the affiliation of Serbian isolates to subgroups has mainly been based on CP gene phylogeny. Thus, data on the viral population structure analyzed at the molecular level, are still lacking in the country. The first preliminary insight into the natural population of CMV in Serbia, covering all five genes, was gained during the development of a reliable RT-PCR-RFLP protocol for the determination of CMV subgroups (Zečević et al., 2023).

To address this knowledge gap, the present study aimed to assess the genetic diversity and population structure of CMV in Serbia, with particular focus on the role of recombination and reassortment in shaping the virus population. To achieve this, we analyzed sequence data from all five open reading frames (ORFs) of nineteen local CMV isolates collected from thirteen different plant species across multiple geographic regions. This study provides the first in-depth molecular characterization of CMV in Serbia and contributes to understanding its evolution and epidemiology within the Western Balkans.

## 2 Materials and methods

### 2.1 Sample collection and virus isolates

To obtain information on the CMV population present in Serbia, samples from various host plants showing symptoms resembling CMV-like infection (**Supplementary Figure S1**) were collected and tested using the DAS-ELISA kit with a commercial antiserum specific for the detection of CMV (Bioreba AG, Switzerland). Out of 189 samples tested, 51 samples (26.98%) were positive for CMV and in total 17 samples were selected for further analysis based on host plant symptoms and origin: six isolates from cucurbits, one from tobacco, two from pepper, three from tomato, one from bean, three from ornamentals, and one from weeds. In addition, two isolates (674–11 and 137-13), previously characterized at the molecular level (Zečević et al., 2023), were also included and used as reference isolates. Metadata regarding the CMV isolates, including their location of origin and the original host plant, are shown in **Table 1**.

**Table 1:** Cucumber mosaic virus isolates originating from various hosts in Serbia.

Isolate	Host	Locality	GenBank accession number				
			CP gene	MP gene	2a gene	2b gene	1a gene
415-07	*Cucurbita pepo* cv. Olinka	Irmovo	KC847072	KT270496	KT270515	KT270534	KT270557
270-09	*Cucurbita pepo* cv. Tosca	Ruma	JX262137	KT270494	KT270513	KT270532	PX394397
201-11	*Cucurbita pepo* cv. Beogradska	Mačkovac	KC847070	KT270491	KT270510	KT270529	PX394396
570-11	*Cucumis sativus*	Debrc	KT270569	KT270500	KT270519	KT270538	KT270551
473-12	*Citrullus lanatus*	Gornji Tavankut	KC878465	KT270497	KT270516	KT270535	KT270549
286-12	*Cucumis melo*	Porodin	KT270566	KT270495	KT270514	KT270533	KT270548
674-11	*Lagenaria siceraria*	Porodin	JX127305	KT270504	KT270523	KT270542	KT270554
650-07	*Nicotiana tabacum*	Bački Petrovac	EU921757	KT270502	KT270521	KT270540	KT270553
723-10	*Capsicum annuum*	Smederevo	KC847075	KT270505	KT270524	KT270543	KT270555
581-11	*Capsicum annuum*	Cekavica	KC414926	KT270501	KT270520	KT270539	KT270552
101-08	*Solanum lycopersicum*	Družetić	KC414925	KT270490	KT270509	KT270528	KT270546
670-08	*Solanum lycopersicum*	Stajkovce	KC847077	KT270503	KT270522	KT270541	KT270558
137-13	*Solanum lycopersicum*	Ub	MH032570	OR257422	OR257420	OR257421	OR257419
367-14	*Solanum lycopersicum*	Porodin	MH032571	PX394395	PX394394	PX394393	PX394392
267-13	*Phaseolus vulgaris*	Vladičin Han	KT270563	KT270493	KT270512	KT270531	KT270547
1-12	*Peperomia tuissana*	Beograd	KC505441	KT270487	KT270506	KT270525	KT270544
540-10	*Wisteria sinensis*	Porodin	KT270568	KT270499	KT270518	KT270537	KT270550
79-13	*Tulipa* sp.	Krnjača	KJ854451	KT270489	KT270508	KT270527	KT270556
58-12	*Stenactis annua*	Beograd	KT270560	KT270488	KT270507	KT270526	KT270545

### 2.2 RNA extraction and RT-PCR amplification

Total RNAs were extracted from 100 mg of symptomatic plant materials using the RNeasy Plant Mini Kit (Qiagen, Germany). To obtain complete or partial fragments of different CMV genomic regions, reverse transcription (RT)-PCR was performed, using the One-Step RT-PCR kit (Qiagen) and previously described primers given in the **Supplementary Table S1** (Zečević et al., 2023). Assays were performed in a reaction mixture of 25 µL total volume, containing 5 µL 5× Qiagen OneStep RT-PCR buffer (containing 12.5 mM MgCl2), 400 µM each dNTPs, 1 µL Enzyme Mix (Omniscript Reverse Transcriptase, Sensiscript Reverse Transcriptase and HotStarTaq DNA Polymerase), and 2 µL extracted RNA. The final primer concentrations were specific for each primer: 1.5 µM RNA1a-fwd, RV11 and CMVCPrev, 3 µM 2brev and CMV3a-rev, 4.5 µM RNA1a-rev and CMVCPfwd, 6 µM RW8, 2bfwd and CMVMP3. Reverse transcription was performed at 50 °C for 30 min, followed by a PCR denaturation step at 95 °C for 15 min, and final extension at 72 °C for 10 min. Cycling conditions and number of cycles were specific for each primer pair (Zečević et al., 2023). Amplicons were analyzed on 1% agarose gel stained with Midori Green Advance DNA Stain (Nippon Genetics Europe, Germany), at 100 V for 45 min and visualized using a UV transilluminator. Additionally, the RT-PCR products of all ORFs from each Serbian isolate were analyzed in five replicates using the previously described RT-PCR-RFLP protocol (Zečević et al., 2023). No mixed reaction patterns were detected in any of the five analyzed ORFs, confirming the absence of mixed infections in the selected Serbian CMV isolates (data not shown).

### 2.3 Sequence analysis, phylogeny, and estimation of population genetic parameters

Products of an expected size for each genomic region/isolate combination, obtained in RT-PCR assays, were purified using a QIAquick PCR Purification Kit (Qiagen) and Sanger sequenced in both directions on an automated sequencer (Macrogen-Europe BV, the Netherlands), using the same primers as for amplifications. For each isolate, nucleotide sequences from both strands of each ORFs were aligned using ClustalW program (Thompson et al., 1994) implemented in MEGA12 software (Kumar et al., 2024), visually inspected and manually edited. Each consensus sequence was verified by BLASTn (Altschul et al., 1990) and deposited in GenBank under the accession numbers listed in **Table 1**.

For each of the five genomic loci, a multiple sequence alignment was generated using the ClustalW program implemented in MEGA12 software and manually trimmed to match the length of the shortest sequence. The sequences for each of the five ORFs (1a, 2a, 2b, MP and CP) were then compared pairwise to estimate genetic diversity by calculating nucleotide (nt) and amino acid (aa) identities using the p-distance model implemented in MEGA12 (Kumar et al., 2024). Single nucleotide polymorphisms (SNPs), defined as positions in the genome where a single nucleotide differs between sequences, were identified for each ORF using DnaSP 6.12.03 (Rozas et al., 2017). SNPs were used to assess genetic variation within and between CMV subgroups, providing insight into the diversity and evolutionary dynamics of the virus population.

DnaSP 6.12.03 was also used to: 1) identify the number of haplotypes (each represented by a set of SNPs inherited as a unit) and haplotype diversity (Hd); 2) analyze fixed differences and shared polymorphisms; and 3) estimate the total number of segregating sites (S), total number of mutations (Eta), nucleotide diversity (π) – average number of nucleotide differences per site between randomly chosen sequences, which accounts for the frequency of polymorphisms, nucleotide polymorphism (θ) – a measure of total diversity based on S, irrespective of the frequency of variants, as well as Tajima’s *D* value to assess deviations from neutrality and infer potential demographic or selective processes shaping genetic variation. Additionally, molecular evolutionary analyses based on the rates of synonymous/non-synonymous changes across the five coding regions were performed using MEGA12 to test the neutrality hypothesis.

To investigate phylogenetic relationships and evolutionary histories at individual loci, five different alignments previously described (1a, 2a, 2b, MP and CP) of Serbian sequences were further aligned with representative strains downloaded from the NCBI database (**Supplementary Table S2**) and phylogenetic trees were constructed using maximum likelihood (ML) method implemented in MEGA12. The best-fit substitution model was selected based on the Bayesian Information Criterion (BIC), and branch support was evaluated using 1,000 ultrafast bootstrap replicates and the SH-like approximate likelihood ratio test (aLRT) with 1,000 replicates. All branches with bootstrap value support <70% were collapsed. RNA sequences from peanut stunt virus strain P (NCBI GenBank accession numbers EU570236, EU570237, and EU570238 for RNA1, RNA2, and RNA3 segments, respectively) were included in the phylogenetic analyses as outgroup.

To assess the potential for genetic exchange through recombination within the Serbian CMV population, a phylogenetic network analysis was performed using SplitsTree 6.4.13 (Huson and Bryant, 2024). For all ORFs, CMV sequences used for phylogeny were aligned, trimmed to the length of the shortest sequenced fragment, and concatenated to obtain sequences of 735, 822, and 1,175 nt for the RNA 1, RNA 2 and RNA 3 segments, respectively. The Neighbor-Net method was applied on concatenated RNA 1, RNA 2 and RNA 3 datasets using default settings, and branch support was evaluated with 1,000 bootstrap replicates.

### 2.4 Recombination analysis

Recombination analyses were performed using RDP v4.56 software package, based on concatenated sequences of the RNA 1, RNA 2, and RNA 3 segments, derived from the corresponding individual loci as explained for splits tree analysis. Possible recombination breakpoints in all sequences were identified using RDP (Martin and Rybicki, 2000), GENECONV (Padidam et al., 1999), BOOTSCAN (Salminen et al., 1995), MAXCHI (Smith, 1992), CHIMAERA (Posada and Crandall, 2001), and SISCAN programs (Gibbs et al., 2000), all implemented in the RDP4 package (Martin et al., 2015), with default parameter settings and a Bonferroni corrected P-value of 0.01. Signals were confirmed as true if recognized by at least four detection methods with a high significance level (P-value of <1.0×10^6^) (Liu et al., 2009). These analyses also determined which sequences were the most similar to the recombinant ones, indicating them as ‘parental isolates’ - lineages from which the analyzed regions of the recombinant genomes likely originated.

## 3 Results

### 3.1 Genetic diversity of Serbian CMV isolates

Nucleotide (nt) and corresponding amino acid (aa) distances between 19 Serbian CMV isolates were examined at all five ORFs. Across all regions (1a, 2a, 2b, MP and CP), nt and aa identities ranged broadly indicating remarkable sequence diversity among Serbian isolates (**Supplementary Table S3**). Region 2b showed the highest nt and aa diversity (63.5% to 100% nt, 43.2% to 100% aa identities), followed by regions 2a, MP, CP, and 1a. Nucleotide distances allowed for the division of isolates into two groups, except in the 1a region, where nt identities suggested the existence of three groups. The classification of isolates, and thus the number of isolates per group, varied depending on the locus examined. Notably, when intragroup nt distances were considered, they remained above 94.9%, indicating much higher sequence identity among isolates within the same group (**Supplementary Table S3**).

To further characterize the genetic variation of the Serbian CMV population, several genetic diversity parameters were calculated (**Table 2**). Measures of genetic variation (such as nucleotide and haplotype diversity), both within subgroups and species-wide (**Table 2**), indicate a high overall level of genetic diversity in the tested Serbian CMV population. Gene 2b exhibited the highest level of genetic variation among all five analyzed regions, as illustrated by Hd and π values of 0.942 and 0.177, respectively (**Table 2**).

**Table 2:** Estimation of genetic diversity between Serbian cucumber mosaic virus isolates based on individual genes and genome-wide, within subgroups and species-wide.

ORF	Subgroup	No. of isolates	Length (bp)	S	Eta	SNP density	No. of haplotypes	Hd	π	θ
1a	IA	11	735	45	45	179.67	11	1.000	0.01885	0.0209
	IB	2	735	6	6	245.00	2	1.000	0.00816	0.00816
	I	13	735	89	93	107.36	13	1.000	0.03769	0.04077
	II	6	735	13	13	339.23	6	1.000	0.00653	0.00775
	population-wide	19	735	188	213	74.28	19	1.000	0.10837	0.08291
2a	IA	17	469	21	21	379.67	10	0.838	0.01288	0.01333
	IB	0	NA	NA	NA	NA	NA	NA	NA	NA
	I	17	469	21	21	379.67	10	0.838	0.01288	0.01333
	II	2	469	9	9	104.22	2	0.871	0.01919	0.01919
	population-wide	19	469	145	151	61.46	12	0.871	0.06718	0.09271
2b	IA	13	340	29	29	152.41	9	0.910	0.02171	0.02806
	IB	0	NA	NA	NA	NA	NA	NA	NA	NA
	I	13	340	29	29	152.41	9	0.910	0.02171	0.02806
	II	6	340	8	8	255.00	5	0.933	0.0086	0.0113
	population-wide	19	340	120	131	53.83	13	0.942	0.17719	0.1237
MP	IA	13	637	22	24	376.41	10	0.923	0.00734	0.0122
	IB	0	NA	NA	NA	NA	NA	NA	NA	NA
	I	13	637	22	24	376.41	10	0.923	0.00734	0.0122
	II	6	637	29	31	131.79	6	1.000	0.01756	0.02141
	population-wide	19	637	163	179	74.25	16	0.965	0.10805	0.08116
CP	IA	12	660	27	27	293.33	11	0.985	0.00909	0.01361
	IB	0	NA	NA	NA	NA	NA	NA	NA	NA
	I	12	660	27	27	293.33	11	0.985	0.00909	0.01361
	II	7	660	12	12	385.00	6	0.958	0.00851	0.00912
	population-wide	19	660	127	133	98.74	17	0.988	0.1093	0.00044
Genome-wide		19	2712	738	804	69.82	19	1.000	0.10823	0.07902

S, total number of segregating sites; Eta, total number of mutations; Haplotype, set of SNPs associated statistically and inherited as a unit; Hd, haplotype diversity; π, nucleotide diversity; θ, mutation rate estimated from S; Genome-wide, concatenated sequences of five ORFs; NA, not applicable.

### 3.2 Global patterns of nucleotide substitutions

With a goal of identifying SNPs, we examined five coding regions representing 2712 nt, in 19 CMV isolates originating from 13 hosts in Serbia, yielding a dataset with over 51500 nt (**Table 2**). Based on nucleotide distances, as well as phylogenetic trees constructed for individual loci (see below), each isolate was classified into one of the two subgroups, IA and II (three subgroups for the 1a gene: IA, IB, and II), which were then treated as populations. Sequences at each locus were compared within and between subgroups to determine levels of intra- and inter-group variation in CMV populations. Analysis of the concatenated dataset revealed 738 SNPs, resulting in an overall SNP density of approximately one per 70 nt, with variation ranging from one per 54 nt in the 2b gene to one per 99 nt in the CP gene. However, the extent of polymorphism within subgroups varied distinctly. At the CP locus, for instance, one SNP per 293 nt and one per 385 nt were detected within subgroups I and II, respectively, illustrating lower level of genetic variation within each subgroup. The degree of polymorphism within subgroups also varied across loci: isolates belonging to subgroup II exhibited less genetic variation at the 1a, 2b, and CP loci, whereas the opposite trend was observed at the 2a and MP loci.

### 3.3 Characterization of haplotypes and phylogenetic relationships

**Figure 1:**
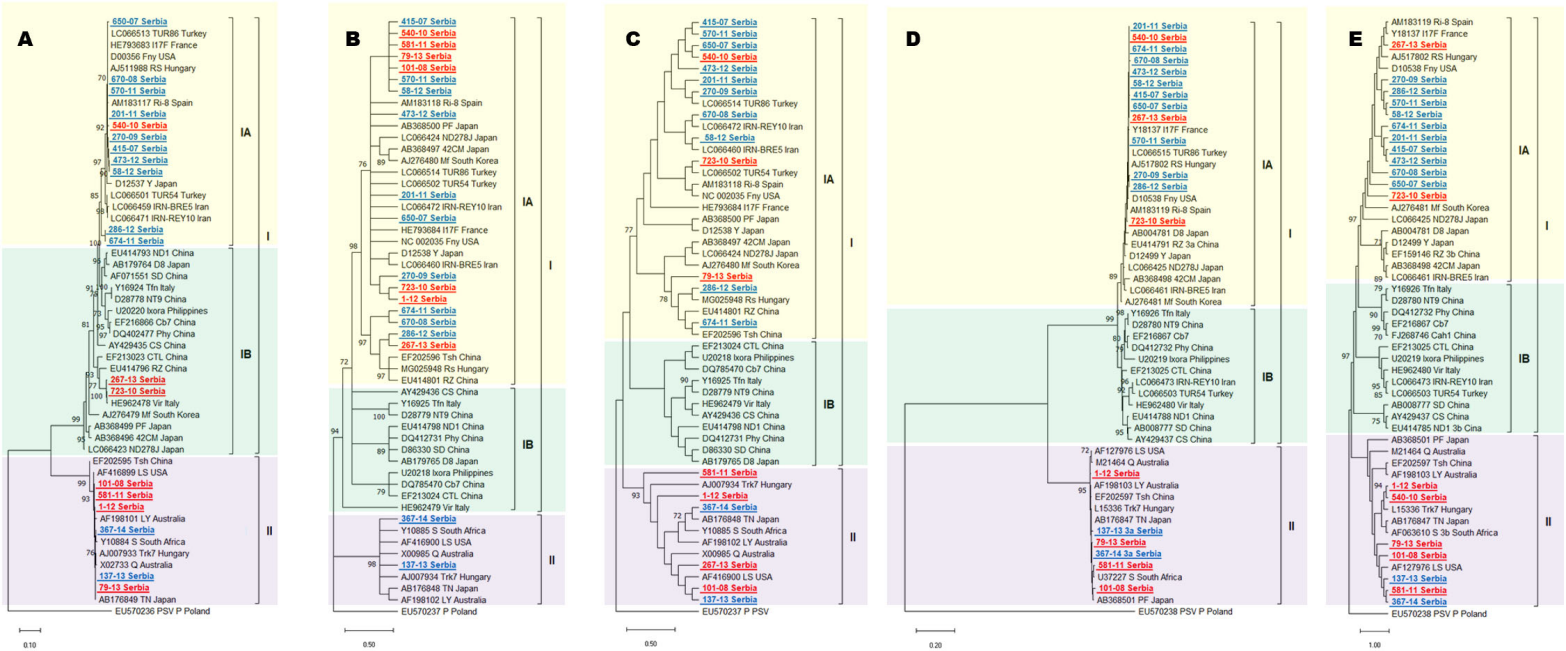
A maximum likelihood phylogenetic tree based on **(A)** partial sequence of the 1a gene; **(B)** partial sequence of the 2a gene; **(C)** complete sequence of the 2b gene; **(D)** partial sequence of the MP gene and **(E)** partial sequence of the CP gene. The tree was constructed using MEGA12 software with the best-fit nucleotide substitution model (T92+G for 1a; K2+G for 2a; K2+G+I for 2b and CP; T92+G+I for MP). Branch support was assessed by bootstrap analysis with 1,000 replicates, and bootstrap values ≥70% are shown next to the corresponding nodes. Cucumber mosaic virus subgroups IA, IB, and II are indicated by different colors (subgroup IA isolates in yellow, subgroup IB isolates in green, and subgroup II isolates in purple). Serbian isolates obtained in this study are shown in bold, underlined, and marked in different colors (blue-isolates with no recombination and reassortant events and red-isolates with recombination and/or reassortant events). Scale bar represents the number of nucleotide changes per sequence.

With the goal of determining the subgroup affiliation of Serbian CMV isolates originating from various hosts and geographic regions, as well as studying evolutionary histories of individual loci, phylogenetic analyses for each of the five ORFs were conducted, based on a comparison with 32 CMV isolates, retrieved from the GenBank and representing different genotypes. Phylogenetic trees constructed via the ML method, based on nucleotide sequences of the 2a, 2b, MP, and CP genes, designated all Serbian CMV isolates as either subgroup IA or subgroup II, with distinct isolate distributions between the two categories depending on the locus (**Figures 1B-E**). For instance, in case of the 2a gene, the vast majority of isolates analyzed in this study (17 out of 19) clustered into subgroup IA, while only two isolates clustered in subgroup II (**Figure 1B**). On the other hand, phylogenetic tree derived from the 1a gene showed that the Serbian CMV isolates separated into three subgroups (IA, IB, and II) (**Figure 1A**). Considering all five phylogenies, these findings are consistent with previously recognized CMV subgroups worldwide and indicate the presence of both subgroup I and II isolates within the population. It is evident that subgroup IA isolates are the most prevalent in the examined Serbian CMV population, while isolates belonging to the IB subgroup were detected only at the 1a gene. It is also important to note that isolates originating from Serbia clustered with international isolates, irrespective of their locations of origin, host, or year sampled.

In addition to the phylogenetic analyses, estimates of DNA divergence (fixed differences and shared mutations between CMV subgroups) were calculated to quantify genetic differentiation among CMV subgroups (**Table 3**). A fixed difference is defined as a nucleotide change that would be present in each sequence from one subgroup but would not be present in the sequences of the other subgroup. A high number of fixed differences and a virtual lack of shared mutations between subgroups I and II (**Table 3**), suggests that the majority of genetic variation observed in the dataset results from the divergence between these two major CMV lineages. Indeed, an examination of isolate-by-isolate fixed differences at individual loci revealed DNA divergence characterized by significantly fewer changes between isolates placed by phylogeny into the same phylogroup and significantly more between sequences originating from distinct phylogroups (**Supplementary Figure S2**). For example, at the 1a locus, 270-09 (IA) and 201-11 (IA) isolate comparison revealed four fixed differences, while 149 fixed polymorphisms were detected between isolates 270-09 (IA) and 1-12 (II) (**Supplementary Figure S2**). Additionally, it should be noted that, within subgroup 1A, isolates 674–11 and 286–12 formed a separate clade from the remaining nine Serbian isolates (**Figure 1A**), which is mirrored in larger number of fixed differences (up to a dozen differences between any pair of the nine isolates from one clade and approximately 30 mutations separating two clades within a subgroup) (**Supplementary Figure S2**). Isolate-by-isolate examination of shared mutations yielded zero shared polymorphisms at all five loci (data not shown). Altogether, this analysis enabled defining isolate- and subgroup- specific mutations in the CMV population.

**Table 3:** DNA divergence between three subgroups of Serbian cucumber mosaic virus population.

ORF	Fixed differences			Shared mutations		
	IA_IB	IA_II	IB_II	IA_IB	IA_II	IB_II
1a	43	129	132	1	0	0
2a	NA	122	NA	NA	1	NA
2b	NA	98	NA	NA	0	NA
MP	NA	126	NA	NA	2	NA
CP	NA	102	NA	NA	1	NA

NA, not applicable.

### 3.4 Examination of putative recombination and reassortment events

**Figure 2:**
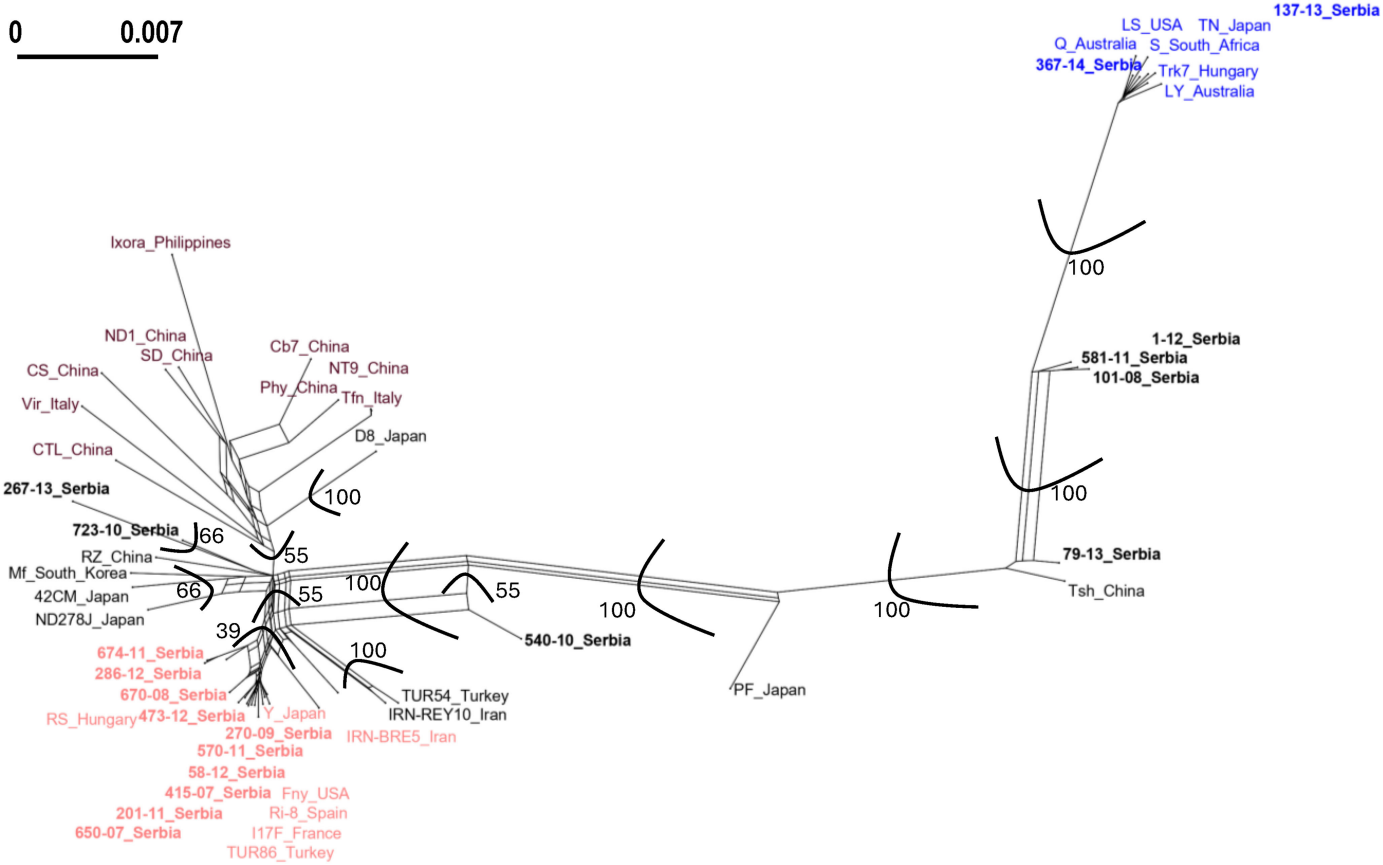
Phylogenetic network of cucumber mosaic virus isolates based on concatenated nucleotide sequences of RNA 1, RNA 2, and RNA 3, constructed using the Neighbor-Net method implemented in SplitsTree 6.4.13. Network-like (reticulate) structures indicate conflicting phylogenetic signals, commonly associated with recombination and/or reassortment events. Bootstrap support was assessed with 1,000 replicates. The scale bar indicates the number of nt substitutions per site and shows how the branch length translates into sequence divergence. The isolates from this study are shown in bold; cucumber mosaic virus subgroups are indicated by different colors: subgroup IA isolates in red, isolates of subgroup IB in burgundy, subgroup II isolates in blue, and recombinants/reassortants in black. Bootstrap support is only given for splits separating main groups.

The phylogenetic network analysis of concatenated sequences of all five genes (1a, 2a, 2b, MP, and CP) showed a reticulate topology, indicating possible recombination and/or reassortment events among the CMV isolates (**Figure 2**). The reticulation patterns confirmed the preliminary observations derived from the comparisons of individual gene phylogenetic trees. Notably, phylogenetic trees based on the 2a and 2b genes (RNA 2) suggested that isolates 101-08, 581-11, 1-12, and 267–13 may be recombinants. These isolates clustered within subgroup IA based on the 2a gene sequence but were placed in subgroup II according to the 2b gene. To further assess the putative recombination signals observed in phylogenetic analyses, we performed recombination detection analyses with the RDP4 program. The program confirmed recombination events in the examined isolates, supported by four to six different detection algorithms and highly significant *P*-values (**Table 4**). Isolates 101-08, 581-11, 1-12, and 267–13 were identified as recombinants between subgroups IA and II in RNA 2, with breakpoints in the 2b gene (positions 464/470–814).

**Table 4:** Summary of recombination events detected among Serbian cucumber mosaic virus isolates based on sequence analyses of individual genomic regions.

RNA segment	RNA 2				RNA 3
Isolate name	101-08	581-11	1-12	267-13	540-10
Score for seven detection methods in RDP4					
RDP	no signal	no signal	7.831×10^-26^	6.639×10^-29^	no signal
GENECONV	6.140×10^-25^	4.708×10^-25^	3.502×10^-24^	3.603×10^-25^	2.592×10^-25^
BootScan	no signal	no signal	no signal	no signal	no signal
MaxChi	1.681×10^-20^	1.952×10^-22^	4.729×10^-21^	2.563×10^-19^	3.009×10^-21^
Chimaera	2.381×10^-20^	1.142×10^-22^	4.757×10^-22^	6.904×10^-20^	4.256×10^-21^
SiScan	no signal	3.694×10^-25^	5.751×10^-24^	1.367×10^-30^	5.842×10^-23^
3Seq	5.695×10^-62^	8.338×10^-57^	1.552×10^-58^	5.670×10^-61^	1.187×10^-60^
Breakpoint position in recombinant sequence					
Begin	470	470	470	470	634
End	814	814	814	814	1172
Parental sequence(s)					
Minor	79-13	Q	Trk7	Tsh	650-07
Major	Trk7	Ri-8	723-10	Trk7	1-12

For each recombinant isolate, the putative parental sequences, genomic breakpoints, and statistical support for recombination are indicated. Recombination signals reflect genetic exchange between different CMV lineages, which may contribute to the emergence of novel viral variants with altered evolutionary and epidemiological properties.

A similar pattern was observed when comparing the phylogenetic trees of the MP and CP genes (RNA 3), indicating a recombination event in isolate 540-10, which grouped within subgroup IA based on the MP gene and within subgroup II based on the CP gene. This isolate was identified as a recombinant between subgroups IA and II within the CP gene (positions 634–1172) by five algorithms implemented in RDP4 (**Table 4**).

In addition to recombination, phylogenetic tree comparisons suggested at least three obvious reassortants among the Serbian isolates included in the current study. In particular, isolate 723–10 grouped within subgroup IB based on the 1a gene, but within subgroup IA for all remaining genes. The second reassortant, isolate 79-13, belonged to subgroup II based on the 1a, MP, and CP genes and to subgroup IA based on the 2a and 2b genes. The recombinant isolate 267–13 also exhibited reassortment event, as it was placed in subgroup IB based on the 1a gene, in subgroup IA based on the 2a, MP and CP genes, and in subgroup II based on the 2b gene.

To assess the genetic diversity of Serbian CMV isolates, each isolate was classified as a genetic variant (e.g., IA-IA, IA-IA, IA) based on the phylogenetic grouping of sequences obtained from three analyzed genomic regions. For the RNA 2 and RNA 3 segments, two sequences corresponding to the genes within each segment were analyzed, and the resulting genotypic assignments are presented sequentially. Overall, based on the phylogenetic grouping of each analyzed region, seven genetic variants were defined within Serbian CMV population: ten isolates (415-07, 650-07, 670-08, 270-09, 201-11, 570-11, 674-11, 58-12, 286–12 and 473-12) possess variant IA-IA,IA-IA,IA; two isolates (137–13 and 367-14) have variant II-II,II-II,II; three isolates (101-08, 581–11 and 1-12) have variant II-IA,II-II,II, while each of the remaining four isolates can be characterized as follows: IB-IA,IA-IA,IA (723-10), IB-IA,II-IA,IA (267-13), IA-IA,IA-IA,II (540-10) and II-IA,IA-II,II (79-13).

### 3.5 Examination of the evolutionary history of the loci

In efforts to learn about the mechanisms responsible for maintaining the levels of genetic diversity observed in the analyzed dataset, we estimated Tajima’s *D*, a statistical test of neutrality under infinite sites-constant population size model. It assesses the relationship between observed (π) and expected (θ) variation and its value can be instrumental in examining evolutionary history of the locus in question. Positive *D* values (a higher variation than expected) can be indicative of balancing selection, population structure or a population decreasing in size, negative *D* values (observed variation is lower than expected) are consistent with positive or negative selection, as well as a population expanding in size. **Table 5** shows Tajima’s *D* results for the five analyzed loci within subgroups I and II, as well as species-wide. Calculated values were negative across all loci within subgroups, although not statistically significant. On the contrary, when Tajima’s *D* values were assessed at the level of an entire population (considering both subgroups together), they were positive in the majority of cases, and statistically significant in the case of the CP gene.

**Table 5:** Measure of selection and population structure, based on individual genes and genome-wide, within subgroups and species-wide.

ORF	Subgroup	Tajima’s *D*	Statistical significance
1a	IA	-0.46243	P > 0.1, NS
	IB	NA*	NA*
	I	-0.34349	P>0.1, NS
	II	-0.95942	P>0.1, NS
	population-wide	1.28903	P>0.1, NS
2a	IA	-0.13541	P>0.1, NS
	IB	NA	NA
	I	-0.13541	P>0.1, NS
	II	NA*	NA*
	population-wide	-1.15158	P>0.1, NS
2b	IA	-0.99063	P > 0.1, NS
	IB	NA	NA
	I	-0.99063	P > 0.1, NS
	II	-1.40833	0.10 > P > 0.05, NS
	population-wide	1.80437	0.10 > P > 0.05, NS
MP	IA	-1.72479	0.10 > P > 0.05, NS
	IB	NA	NA
	I	-1.72479	0.10 > P > 0.05, NS
	II	-0.14106	P > 0.1, NS
	population-wide	1.38817	P > 0.1, NS
CP	IA	-1.48688	P > 0.1, NS
	IB	NA	NA
	I	-1.48688	P > 0.1, NS
	II	-0.363	P > 0.1, NS
	population-wide	2.26390	P < 0.05***
Genome-wide	population-wide	1.08763	P > 0.1, NS

NA*, not applicable, at least 4 sequences necessary for analysis; NA, not applicable, no detected sequences; NS, not significant; ***, statistically significant.

Finally, in attempts to assess selection pressure on CMV coding regions, we estimated rates of synonymous (dS) and nonsynonymous (dN) changes in coding regions and tested for violations of neutrality using a dN/dS (ω) ratio (**Table 6**). Nonsynonymous sites evolving faster than expected (ω>1) are indicative of diversifying (positive) selection, while nonsynonymous sites evolving slower than expected (ω<1) suggest purifying (negative) selection. At all five loci, dN/dS ratios were less than 1.0 (**Table 6**). Gene 1a exhibited the lowest ω values (along with MP and CP genes), approximately five times lower than the 2b locus, perhaps illustrating considerably greater stringency toward amino acid changes.

**Table 6:** Estimation of selection pressure at each locus within the Serbian cucumber mosaic virus population.

ORF	dN^a^	SE^b^	dS^c^	SE	dN/dS^d^
1a	0.04	0.01	0.53	0.07	0.07547
2a	0.05	0.01	0.18	0.03	0.27778
2b	0.18	0.04	0.51	0.14	0.35294
MP	0.05	0.01	0.46	0.08	0.10870
CP	0.05	0.01	0.47	0.08	0.10638

dN, number of ns substitutions per ns site (averaged over all sequence pairs); SE, standard error; dS, number of s substitutions per s site (averaged over all sequence pairs);,dN/dS, calculated by Pamilio-Bianchi-Li (Kumar 2-parameter) substitution method.

## 4 Discussion

The current study represents the first assessment of CMV genetic diversity and population structure in Serbia, across multiple plant hosts and diverse geographic regions, based on sequences of all five ORFs. This work also sheds light, for the first time, on how Serbian isolates relate to global CMV populations.

Our results indicate high level of genetic diversity and the presence of multiple genetic variants, including both recombinant and reassortant isolates. The observed genetic variability reflects a complex and diverse viral population, which may contribute to enhanced adaptability to different environmental conditions. Although this diversity profile contrasts with findings from studies conducted in the USA (Lin et al., 2004; Nouri et al., 2014) and Spain (Fraile et al., 1997; Bonnet et al., 2005), as well as other plant viral populations (García-Arenal et al., 2003), it aligns with more recent data from Nigeria (Apalowo et al., 2022). It is important to note that although the reported extent of diversity is primarily due to genetic differences between subgroups, the observed variability within subgroups may have been overestimated due to the sampling approach. Considering individual loci, based on measures of genetic diversity and SNP density, gene 2b exhibited the highest level of genetic variation among all five analyzed ORFs, which corroborates findings based on American (Lin et al., 2004; Nouri et al., 2014) and Chinese (Liu et al., 2009) CMV populations.

Compared to global patterns of CMV genetic diversity, the Serbian isolates display both shared and unique genomic features. Certain recombination events and subgroup patterns align with reports from other regions, while specific combinations of genomic segments and novel recombinant lineages appear to be region-specific. This suggests that local selective pressures, such as host plant diversity, vector dynamics, and agricultural practices, shape the evolutionary trajectory of the virus in Serbia.

Phylogenetic analyses showed that most Serbian CMV isolates belong to subgroup IA, with the dominant genotype corresponding to the IA-IA,IA-IA,IA variant. This subgroup has been widely recognized as the most predominant and virulent one (Roossinck, 2002; Lin et al., 2004; Liu et al., 2009; Mochizuki and Ohki, 2012; Nouri et al., 2014), although a recent report from Nigeria demonstrated a high frequency of subgroup IB isolates (Apalowo et al., 2022). The presence of group II representatives (variant II-II,II-II,II) was also detected across all loci, but at substantially lower frequencies. These results are consistent with previous studies, which also indicated that both subgroups are present in Serbia, but subgroup IA predominates (Zečević et al., 2023). In addition, subgroup II isolates are considered heat-sensitive and are often found in cooler areas in northern latitudes, representing about 20% of the CMV population on a global scale (Hsu et al., 2000; Bonnet et al., 2005; Davino et al., 2012; Jacquemond, 2012; Ben Tamarzizt et al., 2013). Therewithal, these isolates show less severe symptoms and can often be underestimated (Xu et al., 1999; Tian et al., 2009). Interestingly, no clear association was observed between CMV genetic composition and the host species, geographic origin, or collection year of the Serbian isolates. This lack of correlation, consistently observed across all five genomic regions, is in line with reports from the USA and Asia (Lin et al., 2004; Liu et al., 2009; Nouri et al., 2014). In addition to phylogenetic analyses, examination of DNA divergence between subgroups based on fixed differences was instrumental tool for delineating subgroups, enabling the definition of subgroup-specific mutations.

In attempts to understand mechanisms responsible for sustaining the levels of genetic diversity observed in our dataset, we consider negative values of Tajima’s *D* obtained within all groups at all five loci. Given that the signature of selection typically acts in a locus-specific manner, data seen here for independent loci (excess of low frequency polymorphisms compared to expected within subgroups I and II) suggest demographic effects and are consistent with population size expansion following bottleneck events. These findings corroborate previous reports of recurring population bottlenecks and subsequent founder effects during plant infections (Li and Roossinck, 2004; Lin et al., 2004; Ali and Roossinck, 2010). On the other hand, species-wide positive Tajima’s *D* values indicate population structure and reflect a high degree of DNA divergence between subgroup I and II isolates.

Importantly, consistent with previous reports (Liu et al., 2009; Jacquemond, 2012; Ben Tamarzizt et al., 2013; Kim et al., 2014; Nouri et al., 2014; Hasiów-Jaroszewska et al., 2017, 2018), our results highlight that recombination and reassortment are major evolutionary mechanisms driving the genetic diversity of the CMV population in Serbia. These mechanisms generate novel viral variants, which may enhance adaptive potential, facilitate epidemiological spread, and modulate pathogenicity, complicating disease management by producing strains with altered host range, virulence, or resistance to existing control measures. As previously observed in natural CMV population in Spain (Bonnet et al., 2005), the frequency of recombinants in this study is high, given that the recombination events were identified in both RNA 2 and RNA 3 segments in five of 19 CMV isolates analyzed. Although earlier studies reported a lower frequency of recombination in the RNA 2 segment (Bonnet et al., 2005; Nouri et al., 2014), phylogenetic analyses, together with the recombination signal detected by the RDP4 program, indicated that recombination events occur predominantly within this segment in Serbian CMV isolates. Three isolates (108-08, 581–11 and 1-12) can be categorized as the II-IA,II-II,II variant, while isolate 267–13 represents the IB-IA,II-IA,IA variant, both indicative of recombination in RNA 2. Additionally, recombination in RNA 3 was detected in one isolate (540-10) - the IA-IA,IA-IA,II variant. Although the most common types of recombination in the RNA 3 segment reported thus far have been IA-IB and IB-IA recombinants (Bonnet et al., 2005), besides this study, IA subgroup and subgroup II combinations have also been detected in natural CMV populations in the USA (a recombinant type MP (II)/CP (IA)) (Nouri et al., 2014) and Poland (MP (IA)/CP (II) type) (Hasiów-Jaroszewska et al., 2017).

Genetic exchange through reassortment was detected at a lower frequency (three isolates), which resembles previous reports from the USA (Nouri et al., 2014), while in Tunisia reassortant isolates represented the majority in the natural population (Ben Tamarzizt et al., 2013). Numerous studies (Lin et al., 2004; Bonnet et al., 2005; Ben Tamarzizt et al., 2013; Nouri et al., 2014) have shown that reassortment between subgroups IA and IB is widespread and predominant in different CMV populations worldwide. In contrast, reassortment between subgroups IA/IB and subgroup II is less common, likely due to the heat sensitivity and lower prevalence of subgroup II isolates (Hasiów-Jaroszewska et al., 2018). In this study, both types of reassortment were detected, including reassortment between subgroups IA and IB (isolate 723-10 - the IB-IA,IA-IA,IA variant), as well as IA and II subgroups (isolate 79-13 - the II-IA,IA-II,II variant). So far, the reassortant between subgroups IA and II has been found only in Spain (variant II-II,II-IA,IA) (Bonnet et al., 2005), Japan (variant IA-IA,IA-II,II) (Maoka et al., 2010) and Poland (variant IA-II,II-II,II) (Hasiów-Jaroszewska et al., 2018), while the exact variant observed here (II-IA,IA-II,II) has previously been described only in China (Chen et al., 2007). Moreover, isolate 267-13 (IB-IA,II-IA,IA variant) represents a novel reassortant/recombinant in the natural CMV population. Only one other isolate, which arose from both recombination and reassortment events, has previously been found in the CMV population in Spain (Bonnet et al., 2005). Overall, our findings highlight the critical role of recombination and reassortment in generating new CMV strains. Recognizing these mechanisms is essential for predicting potential outbreaks and developing effective disease management strategies, as the ongoing emergence of novel variants may affect the effectiveness of control measures and the resilience of susceptible crops.

Furthermore, the absence of the IB strain among the isolates examined was notable, except for two reassortants at the 1a gene originating from pepper and bean. Given that their RNA 1 sequence is closely related to the Vir isolate from Italy, we can speculate that they have probably been introduced to Serbia via infected seedlings (as large quantities of tomato seedlings are imported from Italy) but subsequently disappeared from the population due to differences in the fitness of various variants, as proposed by Bonnet et al. (2005). Another possibility is that the IB strain is still present in Serbia, but at such a low frequency that it could not be detected given the number of isolates considered and the experimental design.

Significantly lower levels of genetic diversity and considerably fewer number of fixed differences were detected within subgroups than species-wide, suggesting that most genetic variation arises from divergence between CMV subgroups. Nevertheless, considerable intra-subgroup variation was also detected. For example, isolates 674–11 and 286–12 were characterized by numerous unique mutations and formed a distinct clade from the remaining IA isolates.

Future research should include a more randomized sampling approach, an expanded number of isolates, studies in specific host plants, and cloning of amplicons rather than direct sequencing. These improvements are expected to clarify whether levels of genetic diversity and frequencies of recombinant/reassortant variants observed here accurately reflect the true diversity within Serbian CMV populations or whether they have been overestimated. In addition, such efforts will enable the detection of putative rare genotypes (e.g., low-frequency IB variants) and allow detailed genetic characterization of CMV populations within a single plant.

Despite the fact that CMV is a well-studied plant virus, numerous studies emphasize the necessity to continuously investigate into its diversity, particularly to identify novel mutants, recombinants and reassortants (Fraile et al., 1997; Lin et al., 2004; Bonnet et al., 2005; Chen et al., 2007; Ben Tamarzizt et al., 2013; Nouri et al., 2014). Although hybrid variants could disappear from the population (Bonnet et al., 2005), they can also become established and predominant, as observed in Tunisia (Ben Tamarzizt et al., 2013). Therefore, continued study of CMV genetic variability and the underlying evolutionary mechanisms is essential for understanding and identifying the factors that lead to the emergence and epidemics, ultimately supporting the development of effective and durable disease management strategies.

## Data Availability

The datasets presented in this study can be found in online repositories. The names of the repository/repositories and accession number(s) can be found in the article/**Supplementary Material**.

## Supplementary material

Supplementary Table 1 Primers used for RT-PCR amplification of different cucumber mosaic virus genomic regions.

Supplementary Table 2 Cucumber mosaic virus isolates used for phylogenetic analyses.

Supplementary Table 3 Pairwise nucleotide and amino acid sequence identities among Serbian cucumber mosaic virus isolates within subgroups at five ORFs.

Supplementary Figure 1 Symptoms of cucumber mosaic virus infection: **(A)** – mosaic on *Solanum lycopersicum*; **(B, C)** – mosaic and leaf deformation on *Capsicum annuum*; **(D)** – mosaic and yellowing on *Phaseolus vulgaris*; **(E)** – mosaic on *Cucurbita pepo* cv. Olinka; **(F)** – mosaic and blistering on *Cucurbita pepo* cv. Beogradska; **(G)** – mosaic on *Citrullus lanatus*; **(H)** – mosaic on *Cucumis melo*; **(I)** – fruit deformation on *Cucurbita pepo* cv. Olinka’; **(J)** – mosaic and leaf deformation on *Cucumis melo*; **(K)** – mosaic and leaf deformation on *Stenactis annua*; **(L)** – bright streaks and stripe on leaf on *Tulipa* sp.; **(M)** – mosaic and severe shoestring on *Wisteria sinensis*; **(N, O)** - necrotic ringspots and line patterns on *Peperomia tuisana* leaves.

Supplementary Figure 2 The number of fixed differences between Serbian cucumber mosaic virus isolates based on different ORFs: 1a **(A)**, 2a **(B)**, 2b **(C)**, MP **(D)** and CP **(E).**

## References

[B1] Ali A. Roossinck M. J. (2010). Genetic bottlenecks during systemic movement of cucumber mosaic virus vary in different host plants. Virology 404, 279–283. doi: 10.1016/j.virol.2010.05.017, PMID: 20542533

[B2] Altschul S. F. Gish W. Miller W. Myers E. W. Lipman D. J. (1990). Basic local alignment search tool. J. Mol. Biol. 215, 403–410. doi: 10.1016/S0022-2836(05)80360-2, PMID: 2231712

[B3] Apalowo O. A. Adediji A. O. Balogun O. S. Fakolujo T. I. Archibong J. M. Izuogu N. B. . (2022). Genetic Structure of cucumber mosaic virus from natural hosts in Nigeria reveals high diversity and occurrence of putative novel recombinant strains. Front. Microbiol. 13. doi: 10.3389/fmicb.2022.753054, PMID: 35222322 PMC8866732

[B4] Ben Tamarzizt H. Montarry J. Girardot G. Fakhfakh H. Tepfer M. Jacquemond M. (2013). Cucumber mosaic virus populations in Tunisian pepper crops are mainly composed of virus reassortants with resistance-breaking properties. Plant Pathol. 62, 1415–1428. doi: 10.1111/ppa.12032

[B5] Bonnet J. Fraile A. Sacristán S. Malpica J. M. García-Arenal F. (2005). Role of recombination in the evolution of natural populations of cucumber mosaic virus, a tripartite RNA plant virus. Virology 332, 359–368. doi: 10.1016/j.virol.2004.11.017, PMID: 15661167

[B6] Chen Y. Chen J. Zhang H. Tang X. Du Z. (2007). Molecular evidence and sequence analysis of a natural reassortant between cucumber mosaic virus subgroup IA and II strains. Virus Genes 35, 405–413. doi: 10.1007/s11262-007-0094-z, PMID: 17417698

[B7] Davino S. Panno S. Rangel E. A. Davino M. Bellardi M. G. Rubio L. (2012). Population genetics of cucumber mosaic virus infecting medicinal, aromatic and ornamental plants from northern Italy. Arch. Virol. 157, 739–745. doi: 10.1007/s00705-011-1216-4, PMID: 22218965

[B8] Eiras M. Boari A. J. Colariccio A. Chaves A. L. R. Briones M. R. S. Figueira A. R. . (2004). Characterization of isolates of the Cucumovirus cucumber mosaic virus present in Brazil. J. Plant Pathol. 86, 61–69.

[B9] Fraile A. Alonso-Prados J. L. Aranda M. A. Bernal J. J. Malpica J. M. García-Arenal F. (1997). Genetic exchange by recombination or reassortment is infrequent in natural populations of a tripartite RNA plant virus. J. Virol. 71, 934–940. doi: 10.1128/JVI.71.2.934-940.1997, PMID: 8995610 PMC191141

[B10] Gallitelli D. (2000). The ecology of cucumber mosaic virus and sustainable agriculture. Virus Res. 71, 9–21. doi: 10.1016/s0168-1702(00)00184-2, PMID: 11137158

[B11] García-Arenal F. Fraile A. Malpica J. M. (2003). Variation and evolution of plant virus populations. Int. Microbiol. 6, 225–232. doi: 10.1007/s10123-003-0142-z, PMID: 13680390

[B12] García-Arenal F. Palukaitis P. (2008). “ Cucumber mosaic virus,” in Encyclopedia of virology. Eds. Mahy M. H. V. Regenmortel B.W.J.V. ( Academic Press, Oxford, UK), 614–619. doi: 10.1016/B978-012374410-4.00640-3

[B13] Gaudin J. Piry S. Wipf-Scheibel C. Szadkowski M. Desbiez C. Nguyen E. . (2025). Outbreak of cucumber mosaic virus subgroup ib in pepper from the Espelette area (Basque Country, Southwestern France) and first report of five taxa as natural hosts of CMV. Plant Dis. 109, 983–987. doi: 10.1094/PDIS-07-24-1553-SC, PMID: 39636285

[B14] Giakountis A. Tsarmpopoulos I. Chatzivassiliou E. K. (2018). Cucumber mosaic virus isolates from Greek legumes are associated with satellite RNAs that are necrogenic for tomato. Plant Dis. 102, 2268–2276. doi: 10.1094/PDIS-08-17-1259-RE, PMID: 30189158

[B15] Gibbs M. J. Armstrong J. S. Gibbs A. J. (2000). Sister-scanning: a Monte Carlo procedure for assessing signals in recombinant sequences. Bioinformatics 16, 573–582. doi: 10.1093/bioinformatics/16.7.573, PMID: 11038328

[B16] Gildow F. E. Shah D. A. Sackett W. M. Butzler T. Nault B. A. Fleischer S. J. (2008). Transmission efficiency of cucumber mosaic virus by aphids associated with virus epidemics in snap bean. Phytopathology 98, 1233–1241. doi: 10.1094/PHYTO-98-11-1233, PMID: 18943413

[B17] Hasiów-Jaroszewska B. Budzyńska D. Rymelska N. Korpys P. Borodynko-Filas N. (2018). Phylogenetic evidence of natural reassortants in the cucumber mosaic virus population in Poland. Can. J. Plant Pathol. 40, 587–593. doi: 10.1080/07060661.2018.1509236

[B18] Hasiów-Jaroszewska B. Chrzanowski M. Budzyńska D. Rymelska N. Borodynko-Filas N. (2017). Genetic diversity, distant phylogenetic relationships and the occurrence of recombination events among cucumber mosaic virus isolates from zucchini in Poland. Arch. Virol. 162, 1751–1756. doi: 10.1007/s00705-017-3285-5, PMID: 28238107

[B19] Hirsch J. Moury B. (2021). “ Cucumber mosaic virus (Bromoviridae),” in Encyclopedia of virology, 4th ed. Eds. Bamford D. H. Zuckerman M. ( Academic Press, Oxford, UK), 371–382. doi: 10.1016/B978-0-12-809633-8.21297-1

[B20] Hsu H. T. Barzuna L. Hsu Y. H. Bliss W. Perry K. L. (2000). Identification and subgrouping of cucumber mosaic virus with mouse monoclonal antibodies. Phytopathology 90, 615–620. doi: 10.1094/PHYTO.2000.90.6.615, PMID: 18944541

[B21] Huson D. H. Bryant D. (2024). The SplitsTree app: interactive analysis and visualization using phylogenetic trees and networks. Nat. Methods 21, 1773–1774. doi: 10.1038/s41592-024-02406-3, PMID: 39223398

[B22] Jacquemond M. (2012). Cucumber mosaic virus. Adv. Virus Res. 84, 439–504. doi: 10.1016/B978-0-12-394314-9.00013-0, PMID: 22682176

[B23] Kayode A. B. Odu B. O. Ako-Nai K. A. Alabi O. J. (2014). Occurrence of cucumber mosaic virus subgroups IA and IB isolates in tomatoes in Nigeria. Plant Dis. 98, 1750–1750. doi: 10.1094/PDIS-08-14-0844-PDN, PMID: 30703913

[B24] Kim M. K. Seo J. K. Kwak H. R. Kim J. S. Kim K. H. Cha B. J. . (2014). Molecular genetic analysis of cucumber mosaic virus populations infecting pepper suggests unique patterns of evolution in Korea. Phytopathology 104, 993–1000. doi: 10.1094/PHYTO-10-13-0275-R, PMID: 25116642

[B25] Kumar S. Stecher G. Suleski M. Sanderford M. Sharma S. Tamura K. (2024). MEGA12: Molecular evolutionary genetic analysis version 12 for adaptive and green computing. Mol. Biol. Evol. 41, msae263. doi: 10.1093/molbev/msae263, PMID: 39708372 PMC11683415

[B26] Li H. Roossinck M. J. (2004). Genetic bottlenecks reduce population variation in an experimental RNA virus population. J. Virol. 78, 10582–10587. doi: 10.1128/jvi.78.19.10582-10587.2004, PMID: 15367625 PMC516416

[B27] Lin H. X. Rubio L. Smythe A. B. Falk B. W. (2004). molecular population genetics of cucumber mosaic virus in California: Evidence for founder effects and reassortment. J. Virol. 78, 6666–6675. doi: 10.1128/JVI.78.12.6666-6675.2004, PMID: 15163757 PMC416521

[B28] Lin H. X. Rubio L. Smythe A. B. Jiminez M. Falk B. W. (2003). Genetic diversity and biological variation among California isolates of cucumber mosaic virus. J. Gen. Virol. 84, 249–258. doi: 10.1099/vir.0.18673-0, PMID: 12533721

[B29] Liu Y. Y. Yu S. L. Lan Y. F. Zhang C. L. Hou S. S. Li X. D. . (2009). Molecular variability of five cucumber mosaic virus isolates from China. Acta Virol. 53, 89–97. doi: 10.4149/av_2009_02_89, PMID: 19537909

[B30] Maoka T. Hayano Y. S. Iwasaki M. Yoshida K. Masuta C. (2010). Mixed infection in tomato to ensure frequent generation of a natural reassortant between two subgroups of cucumber mosaic virus. Virus Genes 40, 148–150. doi: 10.1007/s11262-009-0414-6, PMID: 19866353

[B31] Martin D. Rybicki E. (2000). RDP: detection of recombination amongst aligned sequences. Bioinformatics 16, 562–563. doi: 10.1093/bioinformatics/16.6.562, PMID: 10980155

[B32] Martin D. P. Murrell B. Golden M. Khoosal A. Muhire B. (2015). RDP4: Detection and analysis of recombination patterns in virus genomes. Virus Evol. 1, vev003. doi: 10.1093/ve/vev003, PMID: 27774277 PMC5014473

[B33] Milošević D. Stanković I. Ignjatov M. Nikolić Z. Krstić B. (2017). Presence and distribution of pepper viruses in Serbia. Plant Doctor. 45, 647–656.

[B34] Milojević K. Stanković I. Vučurović A. Ristić D. Nikolić D. Bulajić A. . (2013). Biological and molecular characterization of cucumber mosaic virus infecting watermelon in Serbia. Plant Prot. 64, 14–25.

[B35] Mochizuki T. Ohki S. T. (2012). Cucumber mosaic virus: viral genes as virulence determinants. Mol. Plant Pathol. 13, 217–225. doi: 10.1111/j.1364-3703.2011.00749.x, PMID: 21980997 PMC6638793

[B36] Nikolić D. Vučurović A. Stanković I. Radović N. Zečević K. Bulajić A. . (2018). Viruses affecting tomato crops in Serbia. Eur. J. Plant Pathol. 152, 225–235. doi: 10.1093/molbev/msh164, PMID: 15155801

[B37] Nouri S. Arevalo R. Falk B. W. Groves R. L. (2014). Genetic structure and molecular variability of cucumber mosaic virus isolates in the United States. PloS One 9, e96582. doi: 10.1371/journal.pone.0096582, PMID: 24801880 PMC4012352

[B38] Ohshima K. Matsumoto K. Yasaka R. Nishiyama M. Soejima K. Korkmaz S. . (2016). Temporal analysis of reassortment and molecular evolution of cucumber mosaic virus: Extra clues from its segmented genome. Virology 487, 188–197. doi: 10.1016/j.virol.2015.09.024, PMID: 26539800

[B39] Padidam M. Sawyer S. Fauquet C. M. (1999). Possible emergence of new geminiviruses by frequent recombination. Virology 265, 218–225. doi: 10.1006/viro.1999.0056, PMID: 10600594

[B40] Palukaitis P. García-Arenal F. (2003). Cucumoviruses. Adv. Virus Res. 62, 241–323. doi: 10.1016/s0065-3527(03)62005-1, PMID: 14719367

[B41] Palukaitis P. Roossinck M. J. Dietzgen R. G. Francki R. I. B. (1992). Cucumber mosaic virus. Adv. Virus Res. 41, 281–348. doi: 10.1016/s0065-3527(08)60039-1, PMID: 1575085

[B42] Posada D. Crandall K. A. (2001). Evaluation of methods for detecting recombination from DNA sequences: computer simulations. Proc. Natl. Acad. Sci. 98, 13757–13762. doi: 10.1093/oxfordjournals.molbev.a004129, PMID: 11717435 PMC61114

[B43] Rizos H. Gunn L. V. Pares R. D. Gillings M. R. (1992). Differentiation of cucumber mosaic virus isolates using the polymerase chain reaction. J. G. Virol. 73, 2099–2103. doi: 10.1099/0022-1317-73-8-2099, PMID: 1645146

[B44] Roossinck M. J. (2002). Evolutionary history of cucumber mosaic virus deduced by phylogenetic analyses. J. Virol. 76, 3382–3387. doi: 10.1128/jvi.76.7.3382-3387.2002, PMID: 11884564 PMC136033

[B45] Roossinck M. J. Zhang L. Hellwald K. H. (1999). Rearrangements in the 5′ nontranslated region and phylogenetic analyses of cucumber mosaic virus RNA 3 indicate radial evolution of three subgroups. J. Virol. 73, 6752–6758. doi: 10.1128/jvi.73.8.6752-6758.1999, PMID: 10400773 PMC112760

[B46] Rozas J. Ferrer-Mata A. Sánchez-DelBarrio J. C. Guirao-Rico S. Librado P. Ramos-Onsins S. E. . (2017). DnaSP 6: DNA sequence polymorphism analysis of large data sets. Mol. Biol. Evol. 34, 3299–3302. doi: 10.1093/molbev/msx248 29029172

[B47] Salminen M. O. Carr J. K. Burke D. S. Mccutchan F. E. (1995). Identification of breakpoints in intergenotypic recombinants of HIV type 1 by bootscanning. AIDS Res. Hum. Retroviruses 11, 1423–1425. doi: 10.1089/aid.1995.11.1423, PMID: 8573403

[B48] Sclavounos A. P. Voloudakis A. E. Arabatzis C. Kyriakopoulou P. E. (2006). A severe hellenic CMV tomato isolate: symptom variability in tobacco, characterization and discrimination of variants. Eur. J. Plant Pathol. 115, 163–172. doi: 10.1007/s10658-006-0010-8

[B49] Smith J. M. (1992). Analyzing the mosaic structure of genes. J. Mol. Evol. 34, 126–129. doi: 10.1007/BF00182389, PMID: 1556748

[B50] Stanković I. Bulajić A. Vučurović A. Ristić D. Milojević K. Berenji J. . (2011). Status of tobacco viruses in Serbia and molecular characterization of tomato spotted wilt virus isolates. Acta Virol. 55, 337–347. doi: 10.4149/av_2011_04_337, PMID: 22149499

[B51] Stanković I. Vučurović A. Zečević K. Petrović B. Nikolić D. Delibašić G. (2021). Characterization of cucumber mosaic virus and its satellite RNAs associated with tomato lethal necrosis in Serbia. Eur. J. Plant Pathol. 160, 301–313. doi: 10.1007/s10658-021-02241-8

[B52] Thompson J. D. Higgins D. G. Gibson T. J. (1994). CLUSTAL W: Improving the sensitivity of progressive multiple sequence alignment through sequence weighting, position-specific gap penalties and weight matrix choice. Nucleic Acids Res. 22, 4673–4680. doi: 10.1093/nar/22.22.4673, PMID: 7984417 PMC308517

[B53] Tian Z. Qiu J. Yu J. Han C. Liu W. (2009). Competition between cucumber mosaic virus subgroup I and II isolates in tobacco. J. Phytopathol. 157, 457–464. doi: 10.1111/j.1439-0434.2008.01531.x

[B54] Vučurović A. Bulajić A. Stanković I. Ristić D. Berenji J. Krstić B. (2011). Characterization of cucumber mosaic virus originating from cucurbits in Serbia. Pestic. fitomed. 26, 325–336. doi: 10.2298/PIF1104325V

[B55] Wahyuni W. S. Dietzgen R. G. Hanada K. Francki R. I. B. (1992). Serological and biological variation between and within subgroup I and II strains of cucumber mosaic virus. Plant Pathol. 41, 282–297. doi: 10.1111/j.1365-3059.1992.tb02350.x

[B56] Xu P. Li M. Lin Q. Xie L. (1999). Comparative studies on properties of five cucumber mosaic virus isolates infecting Passiflora in China. Virol. Sin. 14, 73–79.

[B57] Zečević K. Stanković I. Petrović B. Krstić B. (2023). Molecular characterization and differentiation of cucumber mosaic virus subgroups in Serbia by RT-PCR-RFLP. Arch. Biol. Sci. 75, 431–342. doi: 10.2298/ABS230718035Z

