# Supplementary material for: Population structure and genetic variation of cucumber mosaic virus isolates in Serbia: evidence for high diversity and the occurrence of natural recombinant and reassortant isolates
Source: Front Plant Sci. 2026 Feb 19;17:1741618. doi: 10.3389/fpls.2026.1741618 (PMC12960582; doi:10.3389/fpls.2026.1741618)
Supplement: Supplementary Table 1 — Primers used for RT-PCR amplification of different cucumber mosaic virus genomic regions. [file DataSheet1.docx]

Supplementary Material

**Supplementary Tabele S1** Primers used for RT-PCR amplification of different cucumber mosaic virus genomic regions

| **ORF** | **Primer name** | **Primer sequence (5′ to 3′)** | **Cycling (temperature/time)** | | | **No. of cycles** | **Amplicon size (bp)** |
| --- | --- | --- | --- | --- | --- | --- | --- |
|  |  |  | **Denaturation** | **Annealing** | **Extension** |  |  |
| 1a | RNA1a-fwd | TGGTAGCCTCCCACGGCGATA | 94°C/60 s | 51°C/60 s | 72°C/60 s | 35 | 1198 |
|  | RNA1a-rev | GAYTGCATRGACATACCATT |  |  |  |  |  |
| 2a | RV11 | GTTTATTTACAAGAGCGTACGG | 94°C/30 s | 53°C/60 s | 72°C/60 s | 35 | 650 |
|  | RW8 | GGTTCGAARRWATAACCGGG |  |  |  |  |  |
| 2b | 2bfwd | TTTGTTGAYMGRYTGAAGTTT | 94°C/60 s | 46°C/60 s 50°C/60 s | 72°C/60 s | 5  30 | 804 |
|  | 2brev | CCTTCCGAAGAAAYCYAGGA |  |  |  |  |  |
| MP | CMVMP3 | GAGTGYGACCTAGGYCGRCATCA | 94°C/60 s | 60°C/60 s | 72°C/60 s | 35 | 728 |
|  | CMV3a-rev | CTAARGACCGTTAACCACCTGC |  |  |  |  |  |
| CP | CMVCPfwd | TGCTTCTCCRCGARWTTGCGT | 94°C/60 s | 52°C/60 s | 72°C/60 s | 35 | 871 |
|  | CMVCPrev | CGTAGCTGGATGGACAACCCG |  |  |  |  |  |

**Supplementary Tabele S2** Cucumber mosaic virus isolates used for phyogenetic analyses

| **Isolate** | **Country** | **Host** | **GenBank Accession Number** | | |
| --- | --- | --- | --- | --- | --- |
|  |  |  | **RNA 1**  **(1a gene)** | **RNA 2**  **(2a and 2b genes)** | **RNA 3**  **(MP and CP genes)** |
| Tfn | Italy | *Solanum lycopersicum* | Y16924 | Y16925 | Y16926 |
| Vir |  | *Capsicum annuum* | HE962478 | HE962479 | HE962480 |
| Ri-8 | Spain | *S. lycopersicum* | AM183117 | AM183118 | AM183119 |
| RS | Hungary | *Raphanus sativus* | AJ511988 | AJ517801 | AJ517802 |
| Trk7 |  | *Trifolium repens* | AJ007933 | AJ007934 | L15336 |
| I17F | France | *S. lycopersicum* | HE793683 | HE793684 | Y18137 |
| FNY | USA | *Cucumis melo* | D00356 | D00355 | D10538 |
| LS |  | *Lactuca sativa* | AF416899 | AF416900 | AF127976 |
| ND1 | China | *Nicotiana tabacum* | EU414793 | EU414798 | EU414788/EU414785 |
| RZ |  |  | EU414796 | EU414801 | EU414791/EF159146 |
| SD |  |  | AF071551 | D86330 | AB008777 |
| Tsh |  | *S. lycopersicum* | EF202595 | EF202596 | EF202597 |
| Cb7 |  |  | EF216866 | DQ785470 | EF216867 |
| NT9 |  |  | D28778 | D28779 | D28780 |
| CS |  | *Arachis hypogaea* | AY429435 | AY429436 | AY429437 |
| CTL |  | *Brassica chinensis* | EF213023 | EF213024 | EF213025 |
| Phy |  | / | DQ402477 | DQ412731 | DQ412732 |
| TN | Japan | *S. lycopersicum* | AB176849 | AB176848 | AB176847 |
| ND278J |  |  | LC066423 | LC066424 | LC066425 |
| PF |  |  | AB368499 | AB368500 | AB368501 |
| D8 |  | *R. sativus* | AB179764 | AB179765 | AB004781 |
| Y |  | *N. tabacum* | D12537 | D12538 | D12499 |
| 42CM |  | *Cucumis sativus* | AB368496 | AB368497 | AB368498 |
| Mf | Korea | *Melandryum firmum* | AJ276479 | AJ276480 | AJ276481 |
| Ixora | Philippines | *S. lycopersicum* | U20220 | U20218 | U20219 |
| Q | Australia | *C. annuum* | X02733 | X00985 | M21464 |
| LY |  | *Lupinus angustifolius* | AF198101 | AF198102 | AF198103 |
| S | South Africa | *Cucurbita pepo* | Y10884 | Y10885 | U37227/AF063610 |
| TUR86 | Turkey | *Rapistrum rugosum* | LC066513 | LC066514 | LC06651 |
| TUR54 |  |  | LC066501 | LC066502 | LC066503 |
| IRN-BRE5 | Iran | *R. sativus* | LC066459 | LC066460 | LC066461 |
| IRN-REY10 |  |  | LC066471 | LC066472 | LC066473 |
| P | Poland | *Lupinus luteus* | EU570236 | EU570237 | EU570238 |

/, unknown host

**Supplementary Table S3** Pairwise nucleotide and amino acid sequence identities among Serbian cucumber mosaic virus isolates within subgroups at five ORFs

| **ORF** | **nt identity** | **aa identity** | **No. of groups** | **nt (aa) identity within subgroups** |
| --- | --- | --- | --- | --- |
| 1a | 79.3-99.9% | 85.7-100% | 3 | group 1 (11 isolates): 95.6-99.9% (98.8-100%) |
|  |  |  |  | group 2 (2 isolates): 99.2% (100%) |
|  |  |  |  | group 3 (6 isolates): 99.2-99.6% (99.2-100%) |
| 2a | 70.1-100% | 50.7-100% | 2 | group 1 (17 isolates): 96.7-100% (93.9-100%) |
|  |  |  |  | group 2 (2 isolates): 98.1% (95.9%) |
| 2b | 63.5-100% | 43.2-100% | 2 | group 1 (13 isolates): 94.9-100% (98.8-100%) |
|  |  |  |  | group 2 (6 isolates): 98.7-100% (97.8-100%) |
| MP | 76.1-100% | 84.7-100% | 2 | group 1 (13 isolates): 97.9-100% (98.6-100%) |
|  |  |  |  | group 2 (6 isolates): 97.2-99.5% (97.6-100%) |
| CP | 76.9-100% | 54.2-100% | 2 | group 1 (12 isolates): 98.0-99.8% (95.1-100%) |
|  |  |  |  | group 2 (7 isolates): 98.8-100% (96.5-100%) |


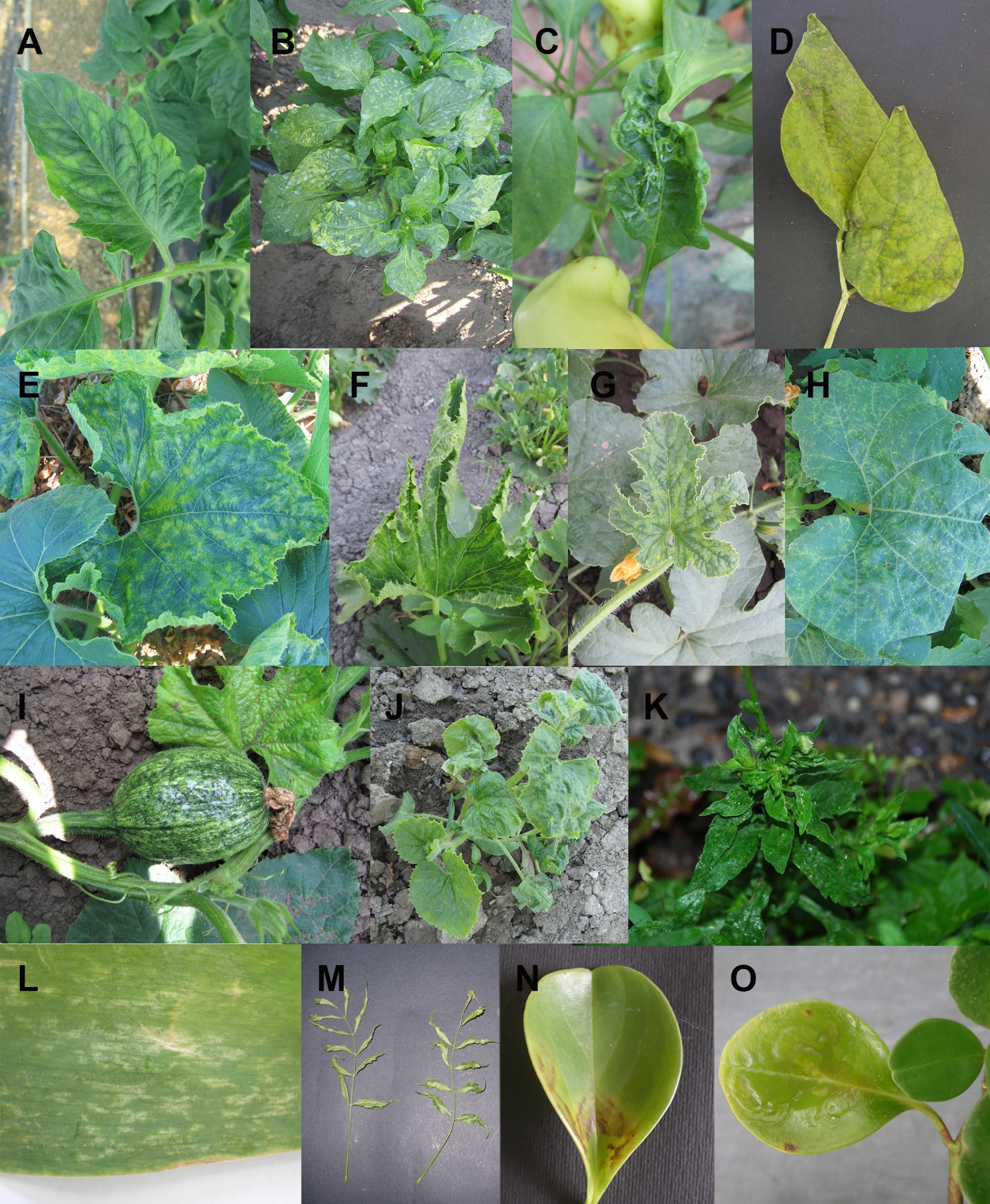


**Supplementary Figure S1** Symptoms of cucumber mosaic virus infection: A – mosaic on Solanum lycopersicum; **B** and **C** – mosaic and leaf deformation on *Capsicum annuum*; **D** – mosaic and yellowing on *Phaseolus vulgaris*; **E** – mosaic on *Cucurbita pepo* cv. Olinka; **F** – mosaic and blistering on *Cucurbita pepo* cv. Beogradska; **G** – mosaic on *Citrullus lanatus*; **H** – mosaic on *Cucumis melo*; **I** – fruit deformation on *Cucurbita pepo* cv. Olinka’; **J** – mosaic and leaf deformation on *Cucumis melo*; **K** – mosaic and leaf deformation on *Stenactis annua*; **L** – bright streaks and stripe on leaf on *Tulipa* sp.; **M** – mosaic and severe shoestring on *Wisteria sinensis*; **N** and **O** - necrotic ringspots and line patterns on *Peperomia tuisana* leaves.


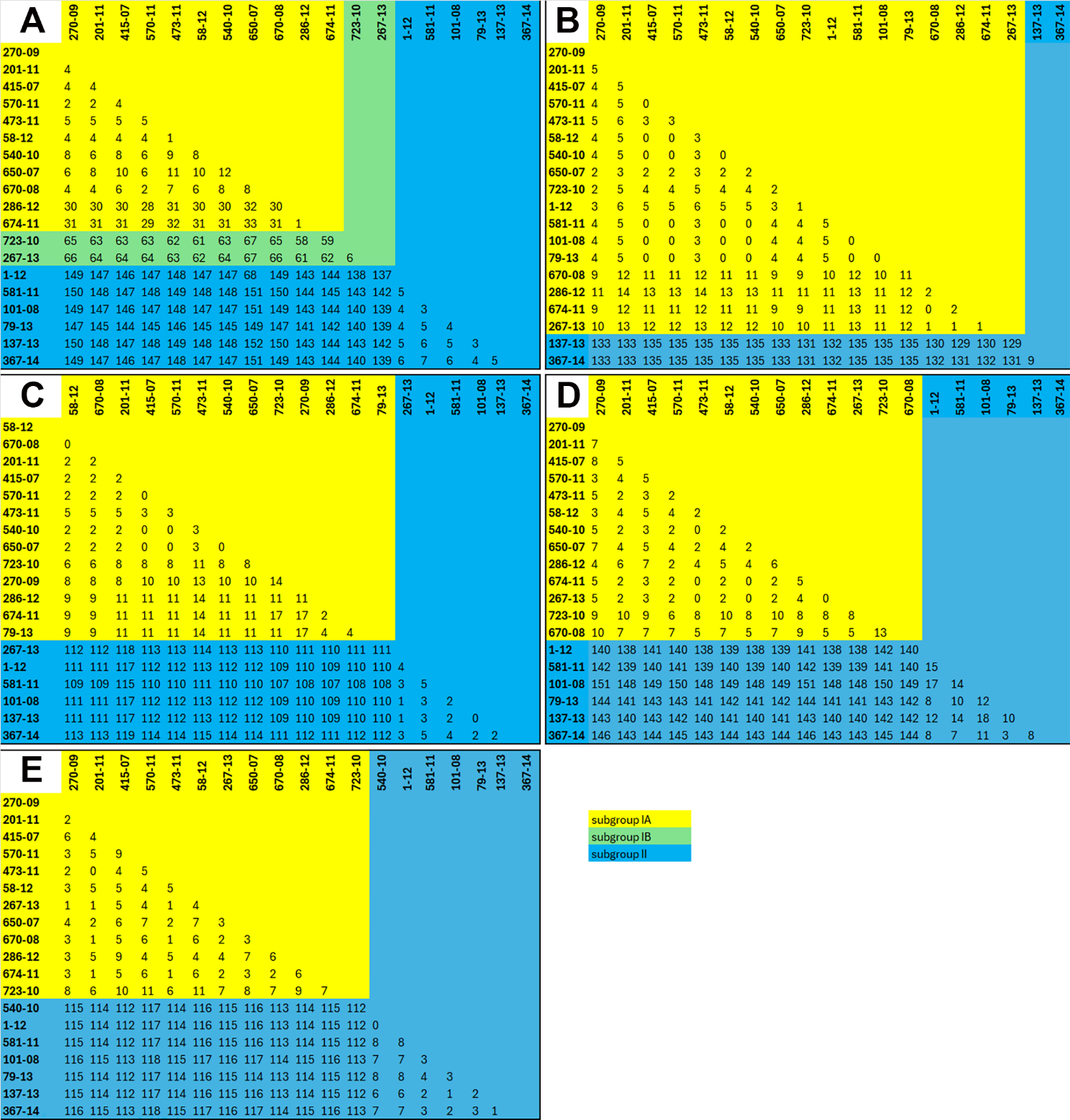


**Supplementary Figure S2** The number of fixed differences between Serbian cucumber mosaic virus isolates based on different ORFs: 1a (A), 2a (B), 2b (C), MP (D) and CP (E)
